# Interest of standardization of feet position during 3-dimensional trunk capture

**DOI:** 10.1186/1748-7161-8-S2-P4

**Published:** 2013-09-18

**Authors:** Gregory Notin, Sophie Pourret, Cyril Lecante, Julie Deceuninck, Nicolas Fraisse, Jean Claude Bernard

**Affiliations:** 1 Lecante, Lyon, France

## Background

Follow-up visits with scoliotic patients require too many radiologic exams. The ORTEN optical sensor[1,2] captures the external shape of the trunk in 3-dimensions that can be used for intermediary exams and limit the usage of x-rays. 

## Purpose

The objective of this study was to demonstrate the value-added benefit of the standardization of the feet posture during the digitization of the patient’s trunk. 

## Methods

a. Bare skin acquisition. b. Cutaneous benchmark: the first sacral piece (S1), the anterior superior iliac spine (ASIS). c. First acquisition in standard position, feet spread natural, hands at shoulder height into vertical bars, elbows drooping. d. Second acquisition in the same position but with feet in a board position with heels spaced 19cm apart[3]. e. A single operator. f. Measuring the angle S1, ASIS right relative to the horizontal and the angle S1, ASIS left relative to the horizontal on the acquisition software Orten file. An average is taken, the angle (S1, ASIS) relative to the horizontal. g. Comparing 24 samples (20 women and 4 men) with two acquisitions. h. Nine samples (7 women and 2 men) are reacquired three days later. 

## Results

a. The angle (S1, EIAS) without platform: from 7.8° to 30°. Average: 16.2° deviation: 22.2°. b. The angle (S1, EIAS) with platform: from 5.95° to 34.7°. Average: 15.9° deviation: 28.75°. c. Difference of -13° to 6.2°. Average: -0.3° deviation: 19.2°. Variations were observed in both directions with an overestimated gap when using the platform. d. Without platform: Difference after three days -9.75° to 10.7°. Average: -1.4° deviation: 20.45°. e. With platform: Difference after three days -8.6° to 18.25°. Average: 1.4° deviation: 26.85°. More variation is observed when the platform is used. 

## Conclusions and discussion

The trend would be to choose the natural position of the feet in order to obtain an exam as reproducible as possible. 
